# Musculoskeletal Fitness Measures Are Not Created Equal: An Assessment of School Children in Corpus Christi, Texas

**DOI:** 10.3389/fpubh.2018.00142

**Published:** 2018-05-22

**Authors:** Toyin Ajisafe, Theresa Garcia, Hsin-Chen Fanchiang

**Affiliations:** ^1^Department of Kinesiology, Texas A&M University, Corpus Christi, TX, United States; ^2^College of Nursing and Health Sciences, Texas A&M University, Corpus Christi, TX, United States; ^3^Department of Physical Education, National Taiwan Sport University, Taoyuan, Taiwan

**Keywords:** obesity, musculoskeletal fitness, elementary school, healthy fitness zone, physical activity

## Abstract

This study investigated current obesity prevalence and associations between musculoskeletal fitness test scores and the odds of being underweight, overweight, or obese compared to having a healthy weight in elementary school children in Corpus Christi, Texas. The sample analyzed consisted of 492 public elementary school children between kindergarten and fifth grade. Their ages ranged from 5 to 11 years. Trunk lift, 90° push-up, curl-up, and back saver sit and reach tests were administered. Weight status was determined using BMI scores and the CDC growth charts. Obesity prevalence remains high among elementary school-aged children in Corpus Christi, Texas. Higher 90° push-up test scores were most consistently associated with decreased odds of being obese as compared to being overweight and having healthy weight except in kindergarten. Conversely, higher trunk lift test scores were associated with increased odds of being obese in second and fourth grades. When children achieved the minimum score to be classified in the Healthy Fitness Zone, those with healthy weight had similarly low musculoskeletal fitness (i.e., abdominal strength and endurance, hamstring flexibility, and trunk extensor strength and flexibility) as peers with overweight and obesity, especially in the lower grades. It was concluded that increased obesity prevalence in higher grades may be precipitated (at least in part) by low musculoskeletal fitness in the lower grades, especially kindergarten. Given previous associations in the literature, low musculoskeletal fitness may be symptomatic of poor motor skill competence in the current sample. These findings suggest a need for early and focused school-based interventions that leverage both known and novel strategies to combat pediatric obesity in Corpus Christi.

## Introduction

Overweight and obesity remains a national priority and a pervasive trend in South Texas. Claims data from Blue Cross and Blue Shield of Texas show high incidence of diabetes with chronic conditions, e.g., chronic kidney disease, in South Texas, including a 107% increase in diabetes in 2016 (1). The incidence of childhood overweight and obesity among school-aged children in Corpus Christi exceeds national rates. Between 2010 and 2013, 37% of fifth graders in Texas had BMI scores associated with adverse health outcomes (2). In 2013, 53.36% of elementary school students assessed using FitnessGram® criteria in Corpus Christi Independent School District were overweight and obese (3). Data from the National Health and Nutrition Examination Survey showed a 35% national prevalence for overweight and obesity among children aged 6 to 11 years between 2007 and 2008 (4). Obesity prevalence was 19.6% between 2007 and 2008, and 17.7% between 2011 and 2012, among the same age group (4, 5).

Obesity is considered a chronic disease resulting from excessive fat accumulation and body mass (6). Children who are obese have increased difficulty controlling their balance compared to their peers with healthy weight (7). This difficulty is thought to discourage them from being physically active or attempting to learn new movements (8). Postural instability is adverse to movement performance (9), and maintaining postural balance is fundamental to many dynamic activities of daily living (7). Two to five year old children with overweight and obesity are more than four times as likely to become overweight and obese adults (10). Hispanic children, including 2–5 year olds, are relatively more affected by overweight and obesity (11). Hispanic-American children were found to be less active at home and during recess at school than non-Hispanic White-American peers (12–15). The case was previously made for early adoption of integrative neuromuscular training, which incorporates fundamental movement skills and activities like plyometric and agility training aimed toward facilitating health- and skill-related fitness (16).

In light of the astounding health- and cost-related impact of obesity (2), Texas passed a senate bill (SB 530) requiring annual health-related physical fitness assessments of public school students in third through twelfth grades in 2007 (17). To accomplish this, the Texas Education Agency adopted FitnessGram® testing (17). FitnessGram® has recommended standards to evaluate and interpret test scores as a reflection of children's positioning on a continuum for health-related fitness (18). Classifications include Healthy Fitness Zone (HFZ), Needs Improvement Zone (NI), and Needs Improvement-Health Risk Zone (NIHRZ). Health-related physical fitness includes musculoskeletal fitness components, namely muscular strength, endurance, and flexibility (18). Upper body, posterior trunk, and abdominal muscle strength and endurance are assessed using the 90° push-up, trunk lift, and curl-up tests, respectively (19). Trunk lift also tests trunk extensor muscle flexibility (19). Hamstring flexibility is assessed using the back saver sit and reach test (19). BMI is recommended as a proxy for body composition, especially where skinfold and bioelectrical impedance analyses are less practical (e.g., in schools) (18).

When optimal, measures of health-related physical fitness are thought to enhance and extend physical activity experience (20). It has been suggested that motor skill competence mediates engagement in physical activity (21) and is variably associated with health-related physical fitness across childhood (i.e., in children aged 4–13 years) (22). Chen et al. (23) concluded that health-related physical fitness components, including trunk lift, push-up, and curl-up tests were linked with students' engagement in physical activity during physical education, recess, and sports outside school. Following equivocal findings on the relationship between pediatric obesity and motor skill development, Castetbon and Andreyeva (8) explored gross motor skills including, skipping a minimum of eight consecutive steps, jumping from a standing start, and hopping unilaterally over five consecutive repetitions. They found that only motor skills that involved vertically displacing the center of mass, namely jumping and hopping, were associated with obesity and standardized BMI scores. Pereira et al. (24) investigated associations between health-related physical fitness and factors, including gross motor coordination, in school-aged Portuguese children. They found that push-up and curl-up tests had the lowest pass rates, while trunk lift test had the highest pass rate (24).

Although the foregoing musculoskeletal fitness measures have been validated and are widely adopted (25), it is unclear whether resulting scores invariably and consistently reflect the odds of being in different weight categories across elementary school age groups in Corpus Christi, Texas. Considering the lack of epidemiological studies and pervasive pediatric obesity in Corpus Christi, it is important to investigate whether elementary school students' performance on current health-related physical fitness assessment criteria reflect the odds of being obese or overweight compared to having healthy weight. Findings will provide a cross-sectional reflection of current overweight and obesity prevalence as well as engender vital discussions on the value of the information from current health fitness zoning approach, particularly with regards to health-related physical fitness.

The aims of the current study were to: (i) investigate whether muscle strength, endurance, and flexibility (i.e., trunk lift, 90° push-up, curl-up, and back saver sit reach test scores) are significantly associated with the odds of being overweight or obese compared to having a healthy weight in elementary school children, (ii) explore relative percentages of children who achieve HFZ classification as a function of weight status across each elementary school grade, and (iii) provide a cross sectional representation of current overweight and obesity prevalence in elementary school-aged children in Corpus Christi, Texas.

## Materials and methods

### Sample

Data were collected from a cross section of 492 public elementary school children in kindergarten, first, second, third, fourth, and fifth grades. Their ages ranged from 5 to 11 years (Table 1A). School demographics consisted of 84.3% Hispanic, 7% African American, 6.7% White, 1.6% Asian, 0.2% American Indian, and 0.2% Two or More races. Ninety three percent of the student population is listed as economically disadvantaged. Fifty nine sets of data were excluded from analysis due to missing fields.

**Table 1A:** Descriptive and anthropometric data.

**Grade**	**Males**	**Females**	**Age (years)**	**Height (m)**	**Weight (kg)**
Kindergarten	39	28	5.55 ± 0.5	1.13 ± 0.05	21.42 ± 4.35
1st Grade	49	31	6.60 ± 0.58	1.19 ± 0.05	24.41 ± 5.95
2nd Grade	30	41	7.67 ± 0.62	1.25 ± 0.07	28.16 ± 7.65
3rd Grade	43	40	8.79 ± 0.57	1.31 ± 0.18	35.29 ± 12.12
4th Grade	42	28	9.63 ± 0.68	1.36 ± 0.25	47.46 ± 25.87
5th Grade	38	30	10.95 ± 0.71	1.42 ± 0.19	44.91 ± 16.44

*Distribution by sex, and mean and standard deviation for age, height, and weight across kindergarten through fifth grade. Kinder is kindergarten*.

A missing field was defined as the absence/omission of an entry for age, weight, height, and actual musculoskeletal fitness test scores, i.e., trunk lift, push-up, curl-up, and back saver sit and reach. Table 1A outlines the average age, height, and weight of the data set from 433 students across six grades. Data was collected under an ongoing agreement with the Corpus Christi independent school district to assess the prevalence of movement-related deficits associated with overweight and obesity among school-aged children. Texas A&M University—Corpus Christi Institutional Review Board approved the study protocol (IRB # 122-17). All subjects gave written informed consent in accordance with the Declaration of Helsinki.

### Procedures

Respective protocols and equipment for trunk lift, push-up, curl-up, and the back saver sit and reach test were implemented as previously described in the FitnessGram® test administration manual (19). Height and weight were measured using a stadiometer and digital scale combination (Tanita, Tokyo, Japan). The same trained resident physical education specialist administered the tests across all the grades. The physical education specialist previously underwent ad hoc training and had previously administered FitnessGram® testing for several years in compliance with the Texas state mandate, Senate Bill 530, which requires testing of school children. The aim of the mandate is to track overweight and obesity and pre-disposition to chronic diseases like type 2 diabetes.

### Data analysis

Participants with any missing data were excluded: one data set was excluded in kindergarten, three were excluded in first grade, 12 were excluded in second grade, three were excluded in third grade, 16 were excluded in fourth grade, and 24 were excluded in fifth grade. Height and weight data were converted from inches and pounds to meters and kilograms, respectively. BMI was computed as the quotient of weight (kg) and the square of height (m). These scores were standardized as z-scores and used to determine respective percentiles for age and sex according to the Centers for Disease Control and Prevention (CDC) growth charts (26). Based on these growth charts (26), underweight, healthy weight, overweight, and obesity were defined as BMI < 5th percentile, 5th ≤ BMI < 85th percentile, 85th ≤ BMI < 95th percentiles, and BMI ≥ 95th percentile, respectively. Given the unequal distances between the percentile-based classifications, the weight classes were treated as categorical data such that underweight was coded as “0,” healthy weight was coded as “1,” overweight was coded as “2,” and obesity was coded as “3.” Although its prevalence is reported, data for underweight participants were excluded from further analysis. The occurrence of underweight in the sample was very low, and its exploration falls outside the scope of current research aims. Measures of musculoskeletal fitness, i.e., muscle strength, endurance, and flexibility, were assessed by the resident physical education specialist at a proxy elementary school in an underserved predominantly Hispanic community in Corpus Christi, Texas. Healthy fitness zoning (HFZ) was done for each student using sex-specific standards specified by FitnessGram®. Scores on trunk lift and the back saver sit and reach tests were measured in inches, while push-up and curl-up were simply the number or repetitions completed.

### Statistical analyses

Data normality was explored using the Shapiro-Wilk significance value on Kolmogorov-Smirnov test of normality. All data were explored for outliers using box plots. Pearson's correlations were calculated to evaluate associations between students' scores on trunk lift, push-ups, curl-ups, and the back saver sit and reach test. This allowed the determination of multicollinearity. A series of multinomial logistic regression analyses were implemented to estimate odd ratios (ORs) and 95% confidence intervals for kindergarten through fifth grade. The healthy weight group was the referent category when exploring odds of being overweight or obese compared to having a healthy weight. A second set of multinomial logistic regression analyses were then implemented switching the referent category to the obese group, in order to explore the odds of being overweight compared to being obese. Although each grade level was analyzed individually, grades were also combined, in order to help account for any effects of sample size on confidence level. Third, fourth, and fifth grades were combined due to increased prevalence of obesity in these grades. Similarly, kindergarten, first, and second grades were combined. Finally, all grade levels were combined, and the same associations were explored. Sex and age were input as covariates in the statistical models, when grades were combined. The magnitudes of associations were presented as ORs and 95% confidence intervals (CIs). Significant two-tailed tests were set at 5% (i.e., *p* < 0.05).

## Results

**Figure 1:**
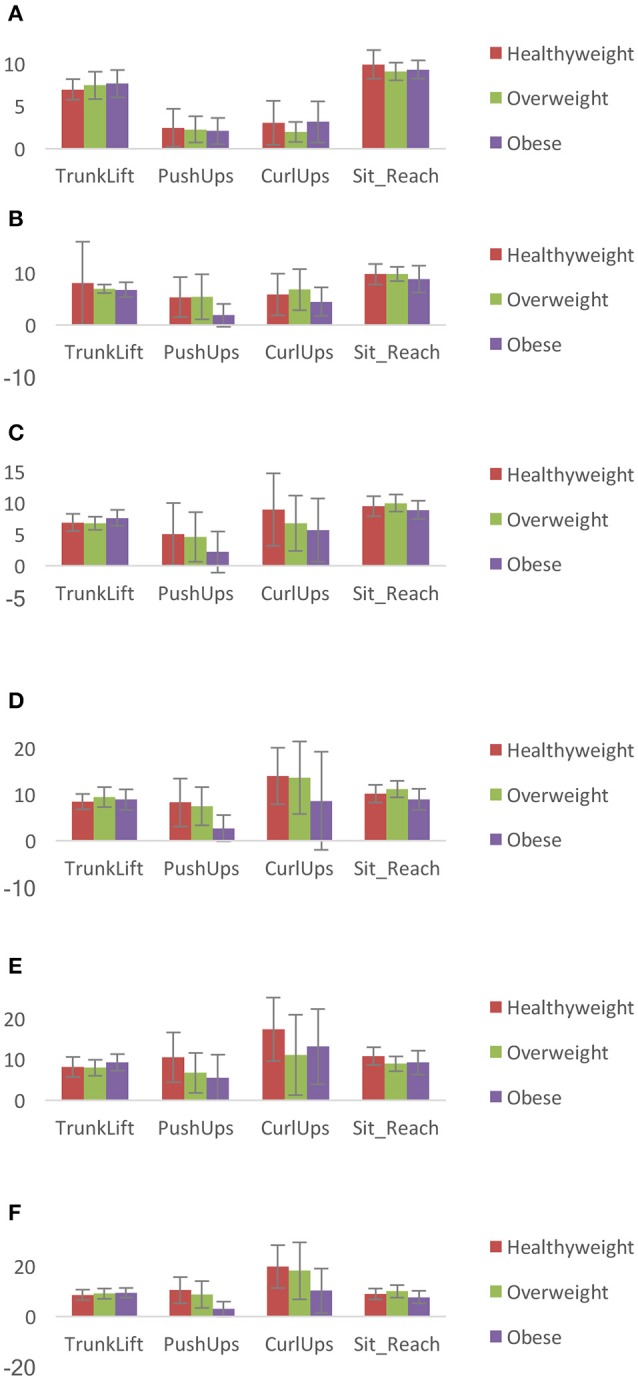
Mean (SD) musculoskeletal fitness scores in: **(A)** kindergarten, **(B)** 1st grade, **(C)** 2nd grade, **(D)** 3rd grade, **(E)** 4th grade, and **(F)** 5th grade. Scores on trunk lift and the back saver sit and reach tests were measured in inches, while push-up and curl-up are the number of repetitions completed.

Thirteen percent of the original 496 data sets was excluded due to missing data field(s). Therefore, 432 (87%) of the original data were deemed intact. The characteristics of the sample considered intact are presented in Table 1A. An additional 17 of the intact data set was excluded from further analysis, because they were classified as underweight. Logistic regression results are presented separately for each grade and for combined grade levels. Mean test scores are displayed in Figure 1.

### Prevalence

The prevalence of overweight in kindergarten, first, second, third, fourth, and fifth grades was 17.6, 8.8, 20.3, 18.1, 11.4, and 17.7%, respectively (Table 1B). The prevalence of obesity in kindergarten, first, second, third, fourth, and fifth grades was 17.6, 26.3, 18.9, 37.3, 55.7, and 33.8%, respectively (Table 1B).

**Table 1B:** Prevalence of each weight category in kindergarten through fifth grade.

**Grade**	**Students**	**Obesity**	**Overweight**	**Healthy**	**Underweight**
		**(%)**	**(%)**	**weight (%)**	**(%)**
Kindergarten	67	17.64	17.64	60.30	4.41
1st Grade	80	26.30	8.80	58.80	6.30
2nd Grade	71	18.92	20.27	43.24	4.05
3rd Grade	83	37.30	18.10	42.20	2.40
4th Grade	70	55.70	11.40	30.00	2.90
5th Grade	62	33.82	17.65	45.59	2.94

### Association between musculoskeletal fitness and weight status in kindergarten

There were no significant, i.e., *p* > 0.05, associations between being overweight, healthy weight or obese, and scores on trunk lift, push-ups, curl-ups, and sit and reach in kindergarten (Tables 2A,B).

**Table 2A:** Fitness associations in kindergarten (reference group: healthy weight).

**Category**	**Variable**	***p*-value**	**Odds ratio**	**95% CI**
Overweight	Trunk lift	0.197	1.372	(0.849, 2.218)
	Push-ups	0.797	1.050	(0.725, 1.521)
	Curl-ups	0.270	0.748	(0.446, 1.253)
	Sit and Reach	0.160	0.677	(0.393, 1.167)
Obese	Trunk lift	0.102	1.482	(0.925, 2.374)
	Push-ups	0.557	0.881	(0.576, 1.346)
	Curl-ups	0.522	1.106	(0.813, 1.504)
	Sit and Reach	0.166	0.691	(0.410, 1.165)

**Table 2B:** Associations between musculoskeletal fitness and weight status in kindergarten (reference group: obese).

**Category**	**Variable**	***p*-value**	**Odds ratio**	**95% CI**
Overweight	Trunk Lift	0.778	0.926	(0.541, 1.585)
	Push-ups	0.500	1.192	(0.716, 1.986)
	Curl-ups	0.172	0.676	(0.386, 1.186)
	Sit and Reach	0.952	0.980	(0.504, 1.906)

### Association between musculoskeletal fitness and weight status in first grade

The only significant associations in first grade were between push-ups (*p* = 0.002; OR = 0.641) and being obese such that the odds of being obese as compared to having healthy weight decreased by 36% for every unit increase in the magnitude of push-ups performed (Table 3A). The odds of being overweight as compared to being obese increased by 47% for every unit increase in the magnitude of push-ups performed (Table 3B). There were no significant associations between trunk lift, push-ups, curl-ups, and being overweight (Tables 3A,B).

**Table 3A:** Associations between musculoskeletal fitness and weight status in first grade (reference group: healthy weight).

**Category**	**Variable**	***p*-value**	**Odds ratio**	**95% CI**
Overweight	Trunk lift	0.741	0.923	(0.575, 1.483)
	Push-ups	0.648	0.943	(0.731, 1.215)
	Curl-ups	0.420	1.116	(0.855, 1.458)
	Sit and Reach	0.761	0.935	(0.606, 1.442)
Obese	Trunk lift	0.350	0.915	(0.760, 1.102)
	Push-ups^*^	0.002	0.641	(0.483, 0.850)
	Curl-ups	0.305	1.112	(0.908, 1.361)
	Sit and Reach	0.096	0.743	(0.523, 1.055)

* *Indicates statistical significance at the level of p < 0.05*.

**Table 3B:** Associations between musculoskeletal fitness and weight status in first grade (reference group: obese).

**Category**	**Variable**	***p*-value**	**Odds ratio**	**95% CI**
Overweight	Trunk Lift	0.974	1.008	(0.614, 1.657)
	Push-ups^*^	0.032	1.470	(1.034, 2.090)
	Curl-ups	0.979	1.004	(0.740, 1.361)
	Sit and Reach	0.372	1.259	(0.759, 2.088)

* *Indicates statistical significance at the level of p < 0.05*.

### Association between musculoskeletal fitness and weight status in second grade

In second grade, there was a significant association between trunk lift (*p* = 0.016; OR = 2.151) and being obese such that the odds of being obese as compared to having healthy weight increased by 115% for every unit increase in the magnitude of trunk lifts performed (Table 4A). The odds of being overweight as compared to being obese weight increased by 107% for every unit increase in the score for the sit and reach (Table 4B). There were no other significant associations between measures of muscle fitness and weight status (Tables 4A,B).

**Table 4A:** Associations between musculoskeletal fitness and weight status in second grade (reference group: healthy weight).

**Category**	**Variable**	***p*-value**	**Odds ratio**	**95% CI**
Overweight	Trunk lift	0.637	0.868	(0.481, 1.565)
	Push-ups	0.482	1.072	(0.883, 1.302)
	Curl-ups	0.224	0.893	(0.744, 1.072)
	Sit and Reach	0.250	1.312	(0.826, 2.084)
Obese	Trunk lift^*^	0.016	2.151	(1.156, 4.001)
	Push-ups	0.167	0.807	(0.595, 1.094)
	Curl-ups	0.715	0.962	(0.784, 1.181)
	Sit and Reach	0.100	0.653	(0.393, 1.086)

* *Indicates statistical significance at the level of p < 0.05*.

**Table 4B:** Associations between musculoskeletal fitness and weight status in second grade (reference group: obese).

**Category**	**Variable**	***p*-value**	**Odds ratio**	**95% CI**
Overweight	Trunk Lift^*^	0.019	0.398	(0.184, 0.862)
	Push-ups	0.085	0.960	(0.963, 1.865)
	Curl-ups	0.527	0.723	(0.752, 1.181)
	Sit and Reach^*^	0.024	2.049	(1.100, 3.817)

* *Indicates statistical significance at the level of p < 0.05*.

### Association between musculoskeletal fitness and weight status in third grade

There was a significant association between push-ups (*p* = 0.000; OR = 0.719) and being obese such that the odds of being obese as compared to having healthy weight decreased by 28% for every unit increase in the magnitude of push-ups performed (Table 5A). The odds of being overweight as compared to being obese increased by 33% for every unit increase in the magnitude of push-ups performed (Table 5B). Similarly, the odds of being overweight as compared to being obese increased by 33% for every unit increase in the sit and reach score (Table 5B). There were no significant associations between trunk lift, curl-ups, or sit-and-reach and weight status when healthy weight was the referent group (Table 5A). There were no significant associations between push-ups, curl-ups, or sit-and-reach and weight status when the referent group was obese (Table 5B).

**Table 5A:** Associations between musculoskeletal fitness and weight status in third grade (reference group: healthy weight).

**Category**	**Variable**	***p*-value**	**Odds ratio**	**95% CI**
Overweight	Trunk lift	0.246	1.249	(0.858, 1.817)
	Push-ups	0.552	0.955	(0.821, 1.111)
	Curl-ups	0.835	0.991	(0.912, 1.077)
	Sit and Reach	0.284	1.210	(0.854, 1.714)
Obese	Trunk lift	0.100	1.374	(0.941, 2.005)
	Push-ups^*^	0.000	0.719	(0.597, 0.866)
	Curl-ups	0.177	0.953	(0.888, 1.022)
	Sit and Reach	0.112	0.725	(0.488, 1.078)

* *Indicates statistical significance at the level of p < 0.05*.

**Table 5B:** Associations between musculoskeletal fitness and weight status in third grade (reference group: obese).

**Category**	**Variable**	***p*-value**	**Odds ratio**	**95% CI**
Overweight	Trunk Lift	0.664	0.909	(0.591, 1.399)
	Push-ups^*^	0.007	1.329	(1.080, 1.635)
	Curl-ups	0.407	1.040	(0.948, 1.142)
	Sit and Reach^*^	0.029	1.668	(1.055, 2.637)

* *Indicates statistical significance at the level of p < 0.05*.

### Association between musculoskeletal fitness and weight status in fourth grade

The only significant associations in fourth grade were between trunk lift (*p* = 0.016; OR = 1.480) and being obese such that the odds of being obese as compared to having healthy weight increased by 48% for every unit increase in the magnitude of trunk lifts performed (Table 6A). The odds of being overweight as compared to being obese increased by 107% for every unit increase in the score for the sit and reach (Table 6B). There were no other significant associations between measures of muscle fitness and weight status when the referent groups were healthy weight and obese (Tables 6A,B, respectively).

**Table 6A:** Associations between musculoskeletal fitness and weight status in fourth grade (reference group: healthy weight).

**Category**	**Variable**	***p*-value**	**Odds ratio**	**95% CI**
Overweight	Trunk lift	0.699	1.091	(0.702, 1.695)
	Push-ups	0.676	0.964	(0.813, 1.144)
	Curl-ups	0.262	0.934	(0.830, 1.052)
	Sit and Reach	0.204	0.794	(0.556, 1.133)
Obese	Trunk lift^*^	0.016	1.480	(1.077, 2.033)
	Push-ups	0.108	0.904	(0.799, 1.022)
	Curl-ups	0.299	0.956	(0.879, 1.040)
	Sit and Reach	0.108	0.804	(0.617, 1.049)

* *Indicates statistical significance at the level of p < 0.05*.

**Table 6B:** Associations between musculoskeletal fitness and weight status in fourth grade (reference group: obese).

**Category**	**Variable**	***p*-value**	**Odds ratio**	**95% CI**
Overweight	Trunk Lift	0.139	0.737	(0.492, 1.104)
	Push-ups	0.442	1.067	(0.905, 1.257)
	Curl-ups	0.673	0.977	(0.877, 1.089)
	Sit and Reach	0.934	0.987	(0.727, 1.341)

### Association between musculoskeletal fitness and weight status in fifth grade

The only significant associations in fifth grade were between push-ups (*p* = 0.003; OR = 0.722) and being obese such that the odds of being obese as compared to having healthy weight decreased by 28% for every unit increase in the magnitude of push-ups performed (Table 7A). The odds of being overweight as compared to being obese increased by 31% for every unit increase in the magnitude of push-ups performed (Table 7B). There were no significant (*p* > 0.05) associations between trunk lift, curl-up, or sit and reach and weight status when healthy weight was the referent group (Table 7A). There were no significant associations between trunk lift, curl-ups, or sit-and-reach and weight status when the referent group was obese (Table 7B).

**Table 7A:** Associations between musculoskeletal fitness and weight status in fifth grade (reference group: healthy weight).

**Category**	**Variable**	***p*-value**	**Odds ratio**	**95% CI**
Overweight	Trunk lift	0.641	1.094	(0.749, 1.599)
	Push-ups	0.499	0.944	(0.800, 1.115)
	Curl-ups	0.491	0.967	(0.879, 1.064)
	Sit and Reach	0.155	1.311	(0.903, 1.905)
Obese	Trunk lift	0.225	1.279	(0.859, 1.904)
	Push-ups^*^	0.003	0.722	(0.583, 0.894)
	Curl-ups	0.572	0.975	(0.891, 1.066)
	Sit and Reach	0.620	0.921	(0.664, 1.276)

* *Indicates statistical significance at the level of p < 0.05*.

**Table 7B:** Associations between musculoskeletal fitness and weight status in fifth grade (reference group: obese).

**Category**	**Variable**	***p*-value**	**Odds ratio**	**95% CI**
Overweight	Trunk Lift	0.493	0.855	(0.547, 1.337)
	Push-ups^*^	0.023	1.307	(1.038, 1.647)
	Curl-ups	0.886	0.992	(0.894, 1.101)
	Sit and Reach	0.097	1.424	(0.938, 2.163)

* *Indicates statistical significance at the level of p < 0.05*.

### Association between musculoskeletal fitness and weight status in third through fifth grades

When third, fourth, and fifth grades were combined (*n* = 183), there was a significant association between push-ups (*p* = 0.000; OR = 0.832) and being obese such that the odds of being obese as compared to having healthy weight decreased by 17% for every unit increase in the magnitude of push-ups performed (Table 8A). The odds of being overweight as compared to being obese increased by 15% for every unit increase in the magnitude of push-ups performed (Table 8B).

**Table 8A:** Associations between musculoskeletal fitness and weight status in third through fifth grades (reference group: healthy weight).

**Category**	**Variable**	***p*-value**	**Odds ratio**	**95% CI**
Overweight	Trunk lift	0.146	1.185	(0.943, 1.488)
	Push-ups	0.305	0.952	(0.867, 1.046)
	Curl-ups	0.255	0.967	(0.913, 1.024)
	Sit and Reach	0.359	1.099	(0.899, 1.343)
Obese	Trunk lift^*^	0.006	1.319	(1.082, 1.606)
	Push-ups^*^	0.000	0.832	(0.762, 0.908)
	Curl-ups	0.278	0.976	(0.934, 1.020)
	Sit and Reach	0.151	0.883	(0.745, 1.047)

* *Indicates statistical significance at the level of p < 0.05*.

**Table 8B:** Associations between musculoskeletal fitness and weight status in third through fifth grades (reference group: obese).

**Category**	**Variable**	***p*-value**	**Odds ratio**	**95% CI**
Overweight	Trunk Lift	0.360	0.898	(0.714, 1.130)
	Push-ups^*^	0.010	1.145	(1.033, 1.269)
	Curl-ups	0.762	0.991	(0.936, 1.050)
	Sit and Reach^*^	0.037	1.244	(1.013, 1.527)

* *Indicates statistical significance at the level of p < 0.05*.

There was a significant association between trunk lift (*p* = 0.006; OR = 1.319) and being obese such that the odds of being obese as compared to having healthy weight increased by 32% for every unit increase in the magnitude of trunk lift performed (Table 8A).

The odds of being overweight as compared to being obese increased by 24% for every unit increase in the sit and reach score (Table 8B). There were no significant associations between curl-ups or sit-and-reach and weight status when healthy weight was the referent group (Table 8A). There were no significant associations between trunk lift or curl-ups and weight status when the referent group was obese (Table 8B). There were no significant differences in the odds of being overweight as compared to having a healthy weight on any of the measures (Table 8A).

### Association between musculoskeletal fitness and weight status in kindergarten through second grades

When kindergarten, first, and second grades were combined (*n* = 214), there was a significant association between push-ups and being obese (*p* = 0.001; OR = 0.764) such that the odds of being obese as compared to having healthy weight decreased by 24% for every unit increase in the magnitude of push-ups performed (Table 9A). The odds of being overweight as compared to being obese increased by 31% for every unit increase in the magnitude of push-ups performed (*p* = 0.006; OR = 1.308) (Table 9B).

**Table 9A:** Associations between musculoskeletal fitness and weight status in kindergarten through second grades (reference group: healthy weight).

**Category**	**Variable**	***p*-value**	**Odds ratio**	**95% CI**
Overweight	Trunk lift	0.694	0.969	(0.831, 1.131)
	Push-ups	0.992	0.999	(0.877, 1.139)
	Curl-ups	0.635	0.972	(0.866, 1.092)
	Sit and Reach	0.825	0.974	(0.772, 1.230)
Obese	Trunk lift	0.536	0.976	(0.904, 1.054)
	Push-ups^*^	0.001	0.764	(0.650, 0.899)
	Curl-ups	0.346	1.055	(0.944, 1.180)
	Sit and Reach^*^	0.029	0.786	(0.633,0.975)

* *Indicates statistical significance at the level of p < 0.05*.

**Table 9B:** Associations between musculoskeletal fitness and weight status in kindergarten through second grades (reference group: obese).

**Category**	**Variable**	***p*-value**	**Odds ratio**	**95% CI**
Overweight	Trunk Lift	0.933	0.993	(0.845, 1.167)
	Push-ups^*^	0.006	1.308	(1.080, 1.583)
	Curl-ups	0.263	0.921	(0.798, 1.063)
	Sit and Reach	0.129	1.239	(0.939, 1.636)

* *Indicates statistical significance at the level of p < 0.05*.

There was a significant association between sit and reach and being obese (*p* = 0.029; OR = 0.786) such that the odds of being obese as compared to having healthy weight decreased by 21% for every unit increase in the magnitude of the sit and reach score (Table 9A). There were no significant differences in the odds of being overweight as compared to having a healthy weight on any of the measures (Table 9A).

### Association between musculoskeletal fitness and weight status in kindergarten through fifth grades

When kindergarten through fifth grades were combined (*n* = 415), there was a significant association between push-ups and being obese (*p* = 0.000; OR = 0.845) such that the odds of being obese as compared to having healthy weight decreased by 16% for every unit increase in the magnitude of push-ups performed (Table 10A). The odds of being overweight as compared to being obese increased by 16% for every unit increase in the magnitude of push-ups performed (*p* = 0.001; OR = 1.157) (Table 10B).

**Table 10A:** Associations between musculoskeletal fitness and weight status in kindergarten through fifth grades (reference group: healthy weight).

**Category**	**Variable**	***p*-value**	**Odds ratio**	**95% CI**
Overweight	Trunk lift	0.326	1.078	(0.928, 1.253)
	Push-ups	0.524	0.978	(0.913, 1.047)
	Curl-ups	0.857	0.996	(0.953, 1.041)
	Sit and Reach	0.678	1.030	(0.896, 1.184)
Obese	Trunk lift^*^	0.009	1.179	(1.042, 1.335)
	Push-ups^*^	0.000	0.845	(0.791, 0.904)
	Curl-ups	0.234	1.022	(0.986, 1.059)
	Sit and Reach^*^	0.001	0.826	(0.736,0.926)

* *Indicates statistical significance at the level of p < 0.05*.

**Table 10B:** Associations between musculoskeletal fitness and weight status in kindergarten through fifth grades (reference group: obese).

**Category**	**Variable**	***p*-value**	**Odds ratio**	**95% CI**
Overweight	Trunk Lift	0.272	0.914	(0.780, 1.073)
	Push-ups^*^	0.001	1.157	(1.064, 1.257)
	Curl-ups	0.290	0.975	(0.929, 1.022)
	Sit and Reach^*^	0.004	1.248	(1.072, 1.452)

* *Indicates statistical significance at the level of p < 0.05*.

There was a significant association between trunk lift (*p* = 0.009; OR = 1.179) and being obese such that the odds of being obese as compared to having healthy weight increased by 18% for every unit increase in the magnitude of trunk lift performed (Table 10A).

There was a significant association between sit and reach and being obese (*p* = 0.001; OR = 0.826) such that the odds of being obese as compared to having healthy weight decreased by 18% for every unit increase in the sit and reach score (Table 10A). The odds of being overweight as compared to being obese increased by 25% for every unit increase in the sit and reach score (Table 10B).There were no significant differences (*p* > 0.05) in the odds of being overweight as compared to having a healthy weight on any of the measures.

## Discussion

This study investigated associations between musculoskeletal fitness scores, i.e., muscle strength, endurance, and flexibility, assessed using trunk lift, 90° push-up, curl-up, and back saver sit and reach, and weight status, in elementary school children in Corpus Christi, Texas. The mean obesity prevalence, across all six grades, of 31.6% observed in the current study, exceeds the previously reported national average of 17.7% among children aged 6–11 years (5). In fact, obesity was consistently more prevalent in this study than the national average except among kindergarteners and second graders where they were comparable (17.64 and 18.92 %, respectively). Similar to previous reports of 37% obesity in fifth grade in Texas (2), 34% of fifth graders in this study were obese.

### Trunk lift test

The lack of associations in kindergarten, first, third, and fifth grades, and the observed pattern of associations in second and fourth grades between trunk lift test scores and the odds of belonging to specific weight categories suggest a pervasive trend: children with overweight and obesity fared just as well or scored higher than peers with healthy weight in some grade levels. Those with overweight did not fare better than those with obesity. Children with obesity had higher trunk lift test scores than those with healthy weight in second and fourth grades (Figures 1C,E). This finding persisted when third, fourth, and fifth grades were combined and when all grade levels were combined. One way to interpret this finding is that being obese did not preclude children from having comparable or in some cases better trunk extensor strength and flexibility than peers with healthy weight. It is plausible that it is easier to be obese and achieve HFZ scoring on the trunk lift test owing to factors such as lower requisite vertical displacement and/or larger physiological cross-sectional area of the muscles involved, compared to other movements in the test battery. Trunk extensors are involved in maintaining an upright trunk. Considering that a child with obesity likely has greater trunk rotational inertia, the resulting increased demand to maintain an upright trunk in the sagittal plane may engender greater relative trunk extensor strength and flexibility.

FitnessGram® standards require scores between 6–12 inches in children aged 5–9 years (i.e., Kindergarten through third grade) and 9–12 inches in children aged 10–12 years (i.e., fourth through fifth grade), in order to achieve HFZ on the trunk lift test. Although upwards of 85% of students achieved HFZ on the trunk lift test across all grades and weight categories except fifth grade (Table 11), scores hovered around the lower end of the standards, even among children with healthy weight. No students achieved the higher ends of the standards for their respective age groups. Mean trunk lift test scores reported by Pereira et al. (24) are more than twice those observed in this study. This suggests children with healthy weight in the current sample did not have stronger or more flexible trunk extensors than peers with overweight and obesity.

**Table 11:** Percentage of children who achieved Healthy Fitness Zone scoring in all grades.

**Grade**	**Trunk lift (%)**	***Push-up* (%)**	**Curl-up (%)**	**Sit and reach (%)**
**KINDERGARTEN**				
Healthy weight	95	39	61	96
Overweight	100	46	55	100
Obese	100	41	83	92
**1ST GRADE**				
Healthy weight	89	72	68	94
Overweight	100	57	86	100
Obese	86	19	67	86
**2ND GRADE**				
Healthy weight	95	39	61	96
Overweight	100	46	55	100
Obese	100	42	83	92
**3RD GRADE**				
Healthy weight	97	69	80	97
Overweight	100	75	62	100
Obese	100	16	35	74
**4TH GRADE**				
Healthy weight	96	82	87	96
Overweight	100	60	60	90
Obese	88	49	71	75
**5TH GRADE**				
Healthy weight	52	81	78	84
Overweight	67	67	67	92
Obese	65	18	39	63

### 90° push-up test

Each unit increase in push-up test scores was associated with decreased odds of being obese in first, third, and fifth grades compared to being overweight and having healthy weight. This finding persisted when the different grade levels were combined This suggests that both the children with healthy weight and overweight fared better than those with obesity in these grades. FitnessGram® standards require scores between 3–8, 4–10, 5–13, 6–15, 7–15 repetitions for children aged 5–6, 7, 8, 9, and 10–11 years, respectively, in order to achieve HFZ on the 90° push-up test. Compared to 75% reported by Chen et al. (23), an average of 66.5% of fifth graders in the current sample achieved HFZ. Only 18% of students with obesity achieved HFZ in fifth grade.

Interestingly, Chen et al. (23) reported an average score of 15 push-ups, while the current study recorded 10 repetitions. Students in the current sample (even when they achieved HFZ) tended to score on the lower end of the HFZ standards (Figure 1). Although the odds were not individually significant possibly due to smaller sample size, the same trend was observed in kindergarten, second, and fourth grades, i.e., children who achieved HFZ scored on the lower end of the FitnessGram® HFZ standards regardless of their weight status. Differences in socioeconomic status and ethnicity may contribute to the tendency toward lower scores in this sample's musculoskeletal fitness test scores. As mentioned earlier, participants in this study included 84% of Hispanic origin and 93% reported as economically disadvantaged (27). Additionally, although there is a dearth of studies specifically reporting the physical activity level of Hispanic children, it has been found that pre-school Hispanic children may have lower levels of physical activity than non-Hispanic White children (12–15). This may be due in part to a lack of access to safe areas to play and exercise in lower income communities (28). The ethnic breakdown and socioeconomic status of the samples in the Chen et al. (18) study, however, were not provided for comparison. Further, unlike trunk extensor muscles, which are involved in maintaining an upright trunk, the primary muscles involved in executing a 90° push-up often have to be deliberately engaged. This engagement commonly occurs during physical activity. Therefore, decreased physical activity levels would expectedly be reflected in upper extremity and shoulder muscle strength.

Test scores were comparable between children with overweight and healthy weight in the current sample and a sample of age-matched Portuguese children (24). This suggests that the 90° push-up test most consistently differentiates between weight status. Castetbon and Andreyeva (8) previously concluded that only gross motor skills that involved translating the body's weight vertically appeared to be inversely associated with obesity. Importantly, there is increased odds of being overweight rather than obese for every increase in push-up score.

### Curl-up test

There were no significant associations between curl-up test scores and the odds of belonging to specific weight categories. FitnessGram® standards require scores between 2–10, 4–14, 6–20, 9–24, 12–24 repetitions for children aged 5–6, 7, 8, 9, and 10–11 years, respectively, in order to achieve HFZ on the curl-up test. It is important to point out that except in third and fifth grades, more than 60% of students who are obese achieved HFZ (Table 8). Previously, an average of 82% of fifth graders recruited from the second year of a three-year intervention project comprising innovative physical education curriculum, mileage club recess, and family community events, achieved HFZ on the curl up test (23). Although a total of 71% of fifth grade students achieved HFZ, only 39% of fifth graders with obesity achieved HFZ on the curl up test in the current sample. Chen et al. (23) did not articulate percentages of HFZ relative to weight status; therefore, the percentage of fifth graders with obesity who achieved HFZ on the curl-up test in their study is unclear.

### Back saver sit and reach test

There were no significant associations between back saver sit and reach test scores and the odds of belonging to specific weight categories. More children with obesity achieved the requisite score for FitnessGram® HFZ on the back saver sit and reach test in kindergarten through second grade than third through fifth grades (Table 8) (18). This may suggest that relative hamstring flexibility deficits emerge in children with obesity after seven years of age. The back saver sit and reach test is especially interesting, because there is no upper limit standard. Unitary scores of 8 inches, for boys, and between 9 and 10 inches, for girls depending on age, are required, in order to achieve HFZ. Therefore, it is challenging to comment on children's performance on a continuum. The lack of associations in kindergarten suggest that children with healthy weight musculoskeletal had just as low scores as those with overweight and obesity. Although obesity was less prevalent in kindergarten, current results may be indicative of poor motor skill competence and inadequate physical activity among in this grade level. Incidentally, Texas schools are not mandated to test children prior to starting the third grade. Considering that children who were overweight and obese between 2 and 5 years of age were more than four times as likely to become overweight and obese adults (10), it is important to address low musculoskeletal fitness among children in kindergarten and pre-kindergarten in Corpus Christi.

### Strengths and limitations

There were several strengths as well as limitations of this study to be noted. The cross-sectional design of the study allowed for inexpensive and very useful analysis of data from a small, but representative sample of the larger population of children in Corpus Christi, Texas. This population of children, 84.3% Hispanic, and 93% economically disadvantaged, well represented the overall high number, 63.5%, of Hispanic people in Corpus Christi, and the median household income of $52,154 (29). This data on FitnessGram® testing and its components' various relationships with child weight categories was not previously available in Corpus Christi, to our knowledge, and thus helps to inform future municipal and state strategies to combat the continuing problem of childhood overweight and obesity in the South Texas area. A further strength of the study is the relative confidence in the accuracy and reliability of the data collected. As described earlier, all data were collected by a single, physical education specialist, trained to collect these data and experienced over several years of FitnessGram® testing cycles.

Limitations of the study findings included the overall small sample size and the high incidence of Hispanic ethnicity and low socioeconomic status in this sample, which decreased generalizability to all populations. This sample, consisting of children from one of the larger elementary schools in the city, was further decreased in size by missing data, consisting primarily of height and weight data, which made it difficult to compare characteristics of the children excluded to those included. Additionally, the rather unexpected finding of a decrease in obesity prevalence from the fourth to the fifth grade cannot be explained by missing data; as stated above, missing data tended to be height and weight measures and there was not a disproportionate number of missing data in the fifth grade that could account for the difference. We theorize that because organized sports often become much more strenuous through elementary and into middle school, increased metabolic demands may have accounted for some of the differences. It is also true that schools in the Corpus Christi area have has been adopting pilot programs focused on district-wide healthy lifestyle initiatives to combat obesity. These initiatives are widely supported by the community and school officials, especially at the elementary school level. Further longitudinal research, including middle and high school data is needed to document the results of these innovative and much needed interventions. Future studies should examine relationships between geographic areas and measures of socioeconomic status, respective park densities and amenities, and average health-related physical fitness measures in schools.

## Conclusions

Overweight and obesity remain highly prevalent among elementary school-aged children in Corpus Christi, Texas. Some children with obesity in the current sample achieved isolated HFZ scoring. Children with healthy weight, especially in the lower grades, tended to have similarly low musculoskeletal fitness as peers with overweight and obesity. It is plausible that spikes observed in obesity prevalence in third through fifth grades are at least in part symptomatic of poor musculoskeletal fitness earlier on, especially in kindergarten. Therefore, greater attention should be paid to the scores themselves, especially among children who score adequately but on the lower end of the HFZ standards. The 90° push test was most consistent at showing significant odds of being obese relative to having a healthy weight. Additional research including a larger and more diverse sample size, longitudinal design, and possibly qualitative data may help further explain the interesting and significant findings of this study.

As noted earlier, there is often a lack of opportunities for physical activity, particularly in economically disadvantaged communities. Since children spend upwards of eight daily hours at school in a presumably safe environment, it would seem optimal that they achieve most, if not all, of the recommended 60 daily minutes of physical activity in that setting. Increasing the amount of physical activity encouraged by the school in classes other than physical education and designing interventions that go beyond the school to actively involve families and communities are broad goals for this community and have shown to be most beneficial in improving healthy lifestyles across the country. Given the association between motor skill competence and health-related physical fitness, it is concluded that there is a need for early and focused school-based interventions that leverage both known and novel strategies to effectively improve motor skill competence and neuromuscular fitness in children in Corpus Christi.

## Data availability statement

Datasets are available on request. The raw data supporting the conclusions of this manuscript will be made available by the authors, without undue reservation, to any qualified researcher.

## Author contributions

TA conceived the study, co-designed the study, performed statistical analyses and interpretation, and co-drafted the manuscript. TG co-designed the study, performed statistical analyses, and co-drafted the manuscript. H-CF performed statistical analyses, and co-drafted the manuscript. All authors have read and approved the final version of the manuscript, and agree with the order of presentation of the authors. TA takes responsibility for the integrity of this work as a whole, from inception to finished article.

### Conflict of interest statement

The authors declare that the research was conducted in the absence of any commercial or financial relationships that could be construed as a potential conflict of interest.

## References

[1] Blue Cross and Blue Shield of Texas Blue Cross and Blue Shield of Texas to Launch $10-million Statewide Initiative to Help Fight Chronic Diabetic Kidney Disease and Chronic Obstructive Pulmonary Disease., Blue Cross and Blue Shield of Texas (2017).

[2] Levitt DE Jackson AW Morrow JR. An analysis of the medical costs of obesity for fifth graders in California and Texas. Int J Exerc Sci. (2016) 9:26–33. Available online at: https://www.ncbi.nlm.nih.gov/pmc/articles/PMC4882466/pdf/ijes-9-1-26.pdf 27293504 10.70252/SCIH7507 PMC4882466

[3] Students Overweight or Obese/Not in Healthy Fitness Zone for Body Composition, Percent by School District, FITNESSGRAM 2012-2013 [Internet]. Community Commons (2013). Available online at: https://maps.communitycommons.org/viewer/

[4] Ogden CL Carroll MD Curtin LR Lamb MM Flegal KM. Prevalence of high body mass index in US children and adolescents, 2007-2008. JAMA (2010) 303:242–9. 10.1001/jama.2009.2012 20071470

[5] Ogden CL Carroll MD Kit BK Flegal KM. Prevalence of childhood and adult obesity in the United States, 2011-2012. JAMA (2014) 311:806–14. 10.1001/jama.2014.732 24570244 PMC4770258

[6] Baselga Torres E Torres-Pradilla M. Cutaneous manifestations in children with diabetes mellitus and obesity. Actas Dermosifiliogr. (2014) 105:546–57. 10.1016/j.ad.2013.11.014 24698434

[7] D'Hondt E Deforche B De Bourdeaudhuij I Lenoir M. Childhood obesity affects fine motor skill performance under different postural constraints. Neurosci Lett. (2008) 440:72–5. 10.1016/j.neulet.2008.05.056 18541379

[8] Castetbon K Andreyeva T. Obesity and motor skills among 4 to 6-year-old children in the united states: nationally-representative surveys. BMC Pediatr. (2012) 12:28. 10.1186/1471-2431-12-28 22420636 PMC3323465

[9] Teasdale N Simoneau M Berrigan F Corbeil P Handrigan GAT Obesity alters balance and movement control. Curr Obes Rep. (2013) 2:235–40. 10.1007/s13679-013-0057-8

[10] Freedman DS Khan LK Serdula MK Dietz WH Srinivasan SR Berenson GS. The relation of childhood BMI to adult adiposity: the Bogalusa Heart Study. Pediatrics (2005) 115:22–7. 10.1542/peds.2004-0220 15629977

[11] Ogden CL Carroll MD Kit BK Flegal KM. Prevalence of obesity and trends in body mass index among US children and adolescents, 1999-2010. JAMA (2012) 307:483–90. 10.1001/jama.2012.40 22253364 PMC6362452

[12] McKenzie TL Sallis JF Nader PR Broyles SL Nelson JA. Anglo- and Mexican-American preschoolers at home and at recess: activity patterns and environmental influences. J Dev Behav Pediatr. (1992) 13:173–80. 1613112

[13] McKenzie TL Sallis JF Elder JP Berry CC Hoy PL Nader PR. Physical activity levels and prompts in young children at recess: a two-year study of a bi-ethnic sample. Res Q Exerc Sport (1997) 68:195–202. 10.1080/02701367.1997.10607998 9294873

[14] O'Connor TM Cerin E Hughes SO Robles J Thompson D Baranowski T. What Hispanic parents do to encourage and discourage 3-5 year old children to be active: a qualitative study using nominal group technique. Int. J. Behav. Nutr. Phys. Act. (2013) 10:93. 10.1186/1479-5868-10-93 23919301 PMC3750326

[15] Ruiz R Gesell SB Buchowski MS Lambert W Barkin SL. The relationship between hispanic parents and their preschool-aged children's physical activity. Pediatrics (2011) 127:888–95. 10.1542/peds.2010-1712 21482607 PMC3387864

[16] Myer GD Faigenbaum AD Ford KR Best TM Bergeron MF Hewett TE. When to initiate integrative neuromuscular training to reduce sports-related injuries and enhance health in youth? Curr Sports Med Rep. (2011) 10:155–66. 10.1249/JSR.0b013e31821b1442 21623307 PMC3105332

[17] Morrow JR Jr, Martin SB, Jackson AW. Reliability and validity of the FITNESSGRAM: quality of teacher-collected health-related fitness surveillance data. Res Q Exerc Sport (2010) 81(3Suppl.):S24–30. 10.1080/02701367.2010.10599691 21049835

[18] Plowman SA Meredith MD Fitnessgram/Activitygram Reference Guide, 4th Edn. Dallas, TX: The Cooper Institute (2013).

[19] Meredith MD Welk G Fitnessgram/Activitygram: Test Administration Manual, 3rd Edn. Champaign, IL: Human Kinetics (2004).

[20] Cattuzzo MT Dos Santos Henrique R Re AH de Oliveira IS Melo BM de Sousa Moura M. Motor competence and health related physical fitness in youth: a systematic review. J Sci Med Sport (2016) 19:123–9. 10.1016/j.jsams.2014.12.004 25554655

[21] Stodden DF Goodway JD Langendorfer SJ Roberton MA Rudisill ME Garcia C A developmental perspective on the role of motor skill competence in physical activity: an emergent relationship. Quest (2008) 60:290–306. 10.1080/00336297.2008.10483582

[22] Stodden DF Gao Z Goodway JD Langendorfer SJ. Dynamic relationships between motor skill competence and health-related fitness in youth. Pediatr Exerc Sci. (2014) 26:231–41. 10.1123/pes.2013-0027 25111159

[23] Chen W Hammond-Bennett A Hypnar A Mason S. Health-related physical fitness and physical activity in elementary school students. BMC Public Health (2018) 18:195. 10.1186/s12889-018-5107-4 29378563 PMC5789625

[24] Pereira SA Seabra AT Silva RG Zhu W Beunen GP Maia JA Correlates of health-related physical fitness levels of Portuguese children. Pediatr Obes. (2011) 6:53–9. 10.3109/17477161003792549

[25] Joshi P Bryan C Howat H. Relationship of body mass index and fitness levels among schoolchildren. J Strength Cond Res. (2012) 26:1006–14. 10.1519/JSC.0b013e31822dd3ac 22371094

[26] Kuczmarski RJ Ogden CL Guo SS Grummer-Strawn LM Flegal KM Mei Z 2000 CDC Growth Charts for the United States: methods and development. Vital Health Stat 11 (2002) 246:1–190. Available online at: https://www.cdc.gov/nchs/data/series/sr_11/sr11_246.pdf 12043359

[27] Texas Education Agency 2015-2016 PFAI Fitness Assessment Data Analysis (2017) [cited 2018]. Available online at: https://tea.texas.gov/Texas_Schools/Safe_and_Healthy_Schools/Physical_Fitness_Assessment_Initiative/Fitness_Data/

[28] Centers for Disease Control and Prevention Physical activity levels among children aged 9-13 years, United States, 2002. MMWR Morb Mortal Wkly Rep. (2003) 52:785–8. Available online at: https://www.cdc.gov/mmwr/preview/mmwrhtml/mm5233a1.htm 12931076

[29] United States Census Bureau 2012-2016 American Community Survey 5-Year Estimates (2016) [cited 2018]. Available online at: https://factfinder.census.gov/faces/nav/jsf/pages/community_facts.xhtml

